# Isodeoxyelephantopin, a Sesquiterpene Lactone Induces ROS Generation, Suppresses NF-κB Activation, Modulates LncRNA Expression and Exhibit Activities Against Breast Cancer

**DOI:** 10.1038/s41598-019-52971-3

**Published:** 2019-11-29

**Authors:** Sumit S. Verma, Vipin Rai, Nikee Awasthee, Anupam Dhasmana, Dhanya S Rajalaksmi, Mangalam S. Nair, Subash C. Gupta

**Affiliations:** 1 Laboratory for Translational Cancer Research, Department of Biochemistry, Institute of Science, Banaras Hindu University, Varanasi, 221 005 India; 2 Swami Rama Himalayan University, Jolly Grant, Dehradun, 248 016 India; 3 Division of Organic Chemistry, CSIR-National Institute for Interdisciplinary Science and Technology, Thiruvanantthapuram, India

**Keywords:** Breast cancer, Breast cancer

## Abstract

The sesquiterpene lactones, Isodeoxyelephantopin (IDET) and Deoxyelephantopin (DET) are known to exhibit activities against some cancer types. The activities of these lactones against breast cancer and the molecular bases is not known. We examined the efficacy of lactones in breast cancer preclinical model. Although both lactones exhibited drug like properties, IDET was relatively effective in comparison to DET. IDET suppressed the proliferation of both invasive and non-invasive breast cancer cell lines. IDET also suppressed the colony formation and migration of breast cancer cells. The assays for Acridine Orange (AO)/Propidium Iodide (PI) staining, cell cycle distribution, phosphatidylserine externalization and DNA laddering suggested the apoptosis inducing potential of IDET. The treatment with IDET also induced an accumulation of cells in the sub-G1 and G2/M phases. The exposure of breast cancer cells to the lactone was associated with a depolarization in mitochondrial membrane potential, and cleavage of caspase and PARP. The lactone induced reactive oxygen species (ROS) generation in breast cancer cells. Further, the use of N-acetyl cysteine (NAC) suppressed IDET induced ROS generation and apoptosis. The NF-κB-p65 nuclear translocation induced by okadaic acid (OA) was suppressed by the sesquiterpene. IDET also suppressed the expression of NF-κB regulated tumorigenic proteins, and induced the expression of proapoptotic gene (Bax) in cancer cells. While the expression of oncogenic lncRNAs was suppressed, the tumor suppressor lncRNAs were induced by the sesquiterpene. Collectively, the modulation of multiple cell signaling molecules by IDET may contribute to its activities in breast cancer cells.

## Introduction

The long non-coding RNAs (lncRNAs) containing equal to more than 200 nucleotides constitute a major class of non-coding RNAs. The lncRNAs play crucial role in multi-steps of tumor development^1^. The preclinical and clinical studies suggest the diagnostic, prognostic and therapeutic potential of lncRNAs^2^. The lncRNAs are often dysregulated in a variety of cancer types including breast cancer^3^.

The lncRNAs can cross talk with other cancer associated molecules such as nuclear factor kappa B (NF-κB), p53, and signal transducer and activator of transcription 3 (STAT3)^4^. Originally identified in the mid-1980s in response to pathogens and viruses^5^, NF-κB is frequently dysregulated in the breast cancer patients. NF-κB is also involved in the development of breast cancer chemoresistance^6^. Under physiological conditions, the heterotrimeric NF-κB (consisting of p65, p50 and IκBα) is localized in the cytoplasm. For NF-κB to be activated, the IκBα must undergo phosphorylation, ubiquitination and degradation. The p65-p50 subunit is then released and translocate from the cytoplasm to the nucleus. NF-κB can modulate multiple cancer related genes and promote the breast cancer growth^7^. During recent years, an association between NF-κB activation pathway and lncRNAs has been reported^8^. Some of the NF-κB associated lncRNAs include antisense non-coding RNA in the INK4 locus (ANRIL)^9^, HOX transcript antisense RNA (HOTAIR)^10^, Lethe^11^, Metastasis Associated Lung Adenocarcinoma Transcript 1 (MALAT1)^12^, NF-κB interacting lncRNAs (NKILA)^13^ and H19^14^.

Because of their crucial role during tumorigenesis, lncRNAs has been targeted therapeutically by the approaches such as antisense oligonucleotides and RNAi technology. However, the studies on the pharmacological intervention of lncRNAs are limited. Since cancer is a multigenic disease, promiscuous drugs with ability to modulate multiple targets are preferred. The agents derived from nature offer potential because of their multi-targeting nature. Approximately 50% of the anticancer drugs approved between 1940 and 2014 were either natural products or drugs derived directly from natural products^15^. *Elephantopus scaber* Linn (family Asteraceae) is a small herb mainly distributed in Africa, Asia, Australia and Europe^16^. The extract from this plant has been shown to exhibit analgesic, anti-asthamatic, anti-diabetic, anti-inflammatory, anti-microbial, anti-oxidant, anti-platelet, hepatoprotective and wound healing activities^17^. The sesquiterpene lactones such as Isodeoxyelephantopin (IDET) and Deoxyelephantopin (DET) are the major constituents from this plant. The sesquiterpenes are known to exhibit activities against colorectal cancer^18^, liver cancer^19^, lung cancer^20^ and nasopharyngeal carcinoma^21^. Previous studies have demonstrated that IDET exhibit activities against some cancer types. However, its potential in breast cancer and the molecular mechanism remains poorly understood. Because breast cancer is an inflammatory disease and IDET is known to exhibit anti-inflammatory activities, our hypothesis in this study was that IDET exhibit activities in breast cancer by modulating inflammatory pathways. A previous study demonstrated that IDET induces cell cycle arrest at G2/M phase in nasopharyngeal carcinoma^21^. In chronic myeloid leukemia cells, IDET can suppress constitutive and inducible NF-κB activation^22^. Conversely, IDET favored lung cancer cell survival through Nrf2-p62-keap1 mediated protective autophagy^20^.

The aim of this study was to examine the anticancer potential of IDET and DET in breast cancer cells. Whether IDET can modulate lncRNAs expression, generation of reactive oxygen species (ROS) and NF-κB activation was also investigated.

## Material and Methods

### Experimental procedures

#### Reagents

IDET and DET was isolated from *Elephantopus scaber* Linn in the laboratory of Dr. Mangalam Nair (CSIR-NIIST, Thiruvananthapuram, India). Doxorubicin hydrochloride was purchased from Tokyo Chemical Industry (Tokoyo, Japan). The trypsin-EDTA, streptomycin, penicillin, Dulbecco’s Modified Eagle’s Medium (DMEM) and N-Acetyl-L-cysteine (NAC) were obtained from Himedia (Mumbai, Maharashtra). The dimethyl sulfoxide (DMSO), crystal violet and 3-[4,5-dimethylthiazol- 2-yl]-2,5-diphenyl tetrazolium bromide (MTT) were purchased from SRL Diagnostics (Mumbai, Maharashtra). Acridine orange; ethidium bromide; propidium iodide; 5,5′,6,6′-Tetrachloro-1,1′,3,3′-tetraethyl benzimidazolyl carbocyanineiodide (JC-1); 4′,6-diamidino-2-phenylindole (DAPI); 2′,7′-dichlorodihydrofluorescein diacetate (H2DCFDA); Alexa Fluor 488; agarose; Annexin V staining kit and fetal bovine serum (FBS) was obtained from Invitrogen (Carlsbad, California). The antibodies for Bcl-xL, Bcl-2, p65, MMP-9 and PARP were obtained from Santa Cruz Biotechnology (Santa Cruz, California). The cleaved caspase 7 and cleaved caspase 9 antibodies were procured from Cell Signaling Technology (Danver, Massachusetts). The primers for cyclinD1, survivin, Bax, ANRIL, lincRNA-Tnfaip3, HOTAIR, GAS5, NKILA, H19 and GAPDH were purchased from Eurofins Genomics (Bangalore, Karnataka). Maxima SYBR Green/ROX qPCR Master Mix (2X) was obtained from Thermo Fisher Scientific (Baltics, Lithuania).

#### Cell lines

We obtained breast cancer cell lines (MDA-MB-468, MDA-MB-453, MDA-MB-231, T47D and MCF-7) from National Centre for Cell Science (NCCS), Pune, India. The cells were cultured in the high glucose DMEM medium. The media was supplemented with FBS (10%), streptomycin (100 µg/mL) and penicillin (100 units/mL).

#### Cell viability assay

The mitochondrial reductase activity^23^ was estimated to examine the effects of IDET and DET on the breast cancer cells viability. In brief, 5,000 cells were seeded in each well of 96 well plate. The cells were then treated with different concentrations of agents for 12–72 hrs. Finally, the formation of purple formazan was measured using MTT as the substrate.

#### Clonogenic assay

We performed an assay as reported before with minor modifications^24^. The cells were first treated with IDET for 24 hrs. The agent was then washed off and the cells were allowed to form colonies. After 7 days, the colonies were stained with crystal violet (0.25%) and counted manually.

#### Live/dead cell discrimination assay

For this assay, we used acridine orange (AO) and propidium iodide (PI) dual staining. AO is permeable to both live and dead cells; it can stain nucleated cells to generate green fluorescence. PI can enter and stain only dead cells with compromised membrane integrity to generate red fluorescence. Briefly, cells (MCF-7 and MDA-MB-231) were treated with 5–25 µM IDET for 24 hrs, washed and stained with AO/PI (100 µg/mL). Finally, we examined the stained cells under fluorescence microscope.

#### Phosphatidylserine externalization assay

A marker for the early phases of apoptosis is the externalization of phosphatidylserine (PS) from the inner surface of the plasma membrane to the outer surface. This results in the disruption of membrane symmetry. For this, we used annexin V/PI staining and followed a method as provided by the supplier (Invitrogen).

#### DNA laddering assay

DNA laddering wherein nuclear DNA undergo fragmentation is one of the key features of apoptosis. This assay was performed using a previously described method with minor modifications^25^. In brief, the cell lysis was performed at 37 °C for 30 min in a buffer consisting of 20 mM EDTA, 100 mM Tris (pH 8.0), RNaseA (500 unit/ml), and 0.8% SDS. The cell lysate was partially deproteinized with proteinase k (20 mg/mL) for 2 hrs at 55 °C. The DNA was precipitated with chloroform and isopropanol. After washing with 70% ethanol and air drying, Tris-EDTA buffer was used to dissolve the DNA. The agarose gel (1.5%) containing ethidium bromide was used to electrophorese the DNA. Finally, the gel documentation (BioRad Gel Doc XR+) was used for the visualization and imaging of DNA bands.

#### Cell cycle analysis

The cells were stained with PI to examine if IDET affects different cell cycle phases. After treatment with various concentrations of IDET, the cells were washed with PBS and fixed with 70% chilled methanol. The RNaseA was used for the treatment of cells followed by staining with PI. We used flow cytometer to assess the percentage of cells and the Cell Quest software (Becton Dickinson) for the analysis.

#### Measurement of mitochondrial membrane potential (ΔΨ)

The effects of IDET on mitochondrial membrane potential was examined using a previously described method^26^. In brief, cells were exposed to 10–50 μM IDET, washed and incubated in the dark (at 37 °C for 20 minutes) with 10 μg/mL JC-1. Cells were then washed and imaged under fluorescence microscopy. Whereas green fluorescence is an indicator of depolarized mitochondria, intact mitochondria produce red fluorescence.

#### Western blot analysis

The western blot analysis was performed to examine the effects of IDET on the expression of tumorigenic proteins^27^. Briefly, the whole cell lysate was prepared from normal and IDET treated cells. After separation on the SDS-PAGE and transferring onto nitrocellulose membrane, the proteins were probed with primary and secondary antibodies. Finally, the ECL reagent was used for the detection of the protein signals.

#### Immunocytochemistry for the NF-κB p65 cellular localization

For this, we used a previously described method^27^. Briefly, paraformaldehyde and PBST were used for the fixing and permeabilization of the cells, respectively. After probing with antibodies (primary and secondary) and counterstaining with DAPI, the cells were imaged under fluorescence microscope.

#### Cell migration assay

Whether IDET affects cell migration was examined by scratch (wound healing) assay^28^. Briefly, at the 70% confluency, the monolayer cells were wounded with a sterile culture tip. After washing the debris, IDET was applied over cells. The wounded area was examined at 0, 9, 24 and 48 hrs by phase contrast microscope. The image J software was used to calculate the healed area and wound size at each time point.

#### Estimation for ROS generation

The potential of IDET to generate ROS in breast cancer cells was examined by flow cytometry^29^. Briefly, the control and treated cells were stained with 10 µM H2DCFDA for 1 hr in the dark. We used flow cytometry to examine the stained cells and Cell Quest software (Becton Dickinson) for the data analysis.

#### Semi-quantitative and quantitative RT-PCR

We performed semiquantitative RT-PCR to examine the IDET’s effects on the expression of mRNA transcripts of cyclin D1, survivin and Bax. The quantitative real-time PCR was performed to examine the IDET’s effects on the lncRNA expression^30^. Table 1 lists the primer sequences used for the amplification of the gene of interest.

**Table 1:** The sequences of the primers used in the semi-quantitative and quantitative RT-PCR.

Gene/lncRNA	Forward sequence (5′-3′)	Reverse sequence (5′-3′)
**Semi-quantitative RT-PCR**		
Bax	CCAAGAAGCTGAGCGAGTGT	CCGGAGGAAGTCCAATGTC
Cyclin D1	CTCCACCTCACCCCCTAAAT	AGAGCCCAAAAGCCATCC
Survivin	GACACTTAGTATGGGAGGGTTG	ACCAAGGCACCAGCATATAG
GAPDH	GCTCTCTGCTCCTCCTGTTC	ACGACCAAATCCGTTGACTC
**Quantitative RT-PCR**		
H19	ATCGGTGCCTCAGCGTTCGG	CTGTCCTCGCCGTCACACCG
GAS5	CTTCTGGGCTCAAGTGATCCT	TTGTGCCATGAGACTCCATCAG
NKILA	TGGATTGTTGGGTATATTTTGGA	TGTATGAAGAGGATGCTGAAGGC
lincRNA-Tnfaip3	GGCTCAGTTGCCATAGAGACTC	CCCACAGCCTACCAAACATC
ANRIL	TGCTCTATCCGCCAATCAGG	GGGCCTCAGTGGCACATACC
HOTAIR	GGTAGAAAAAGCAACCACGAAGC	ACATAAACCTCTGTCTGTGAGTGCC
ACTB	CTGTGGCATCCACGAAAC	CAGACAGCACTGTGTTGG
5Sr RNA	GGCCATACCACCCTGAACGC	CAGCACCCGGTATTCCCAGG

The trizol reagent was used for the isolation of total RNA by following the manufacturer’s instructions (Invitrogen). The high capacity cDNA synthesis kit was used for reverse transcription.

We used 1.5% agarose gel for the electrophoresis of the PCR product. The densitometry and ImageJ software were used for the quantification of DNA bands. The PCR product for the gene of interest were normalized to GAPDH. The Maxima SYBR Green/ROX qPCR Master Mix and Applied Biosystems 7500 Real-Time system was used for the quantitative real-time PCR analyses of lncRNA expression. The data analysis was performed using a method as described previously^31^. The values for house-keeping genes (ACTB and 5SrRNA) were used for the normalization of the data.

#### *In silico* analysis

The drug like properties of IDET (Pub Chem ID: 38359583) and DET (Pub Chem ID: 6325056) were examined by analyzing Lipinski’s rule of five (http://www.molinspiration.com//cgi-bin/properties) and ADMET (absorption, distribution, metabolism, excretion and toxicity)^32^. SMILE IDs of IDET and DET were obtained from Pub Chem database. CORINA 3D server was used to convert the SMILE ID to .pdb files. PDB ID 1NFI was used to procure 3D structure of NF-κB-p65 (Chain A), NF-κB-p50 (Chain B) and IκBα (Chain E). 2EVA and 5TQY was used to procure TAK-1 and IKKα, respectively. Auto Dock Tool 4 was used for the identification of binding affinities and poses of ligands and proteins^33,34^.

### Statistical analysis

Different end points were performed in control and treated groups. We performed the unpaired Student’s t-test for the comparison between the two groups. Statistical significance was calculated at a value of P < 0.05.

## Results

**Figure 1:**
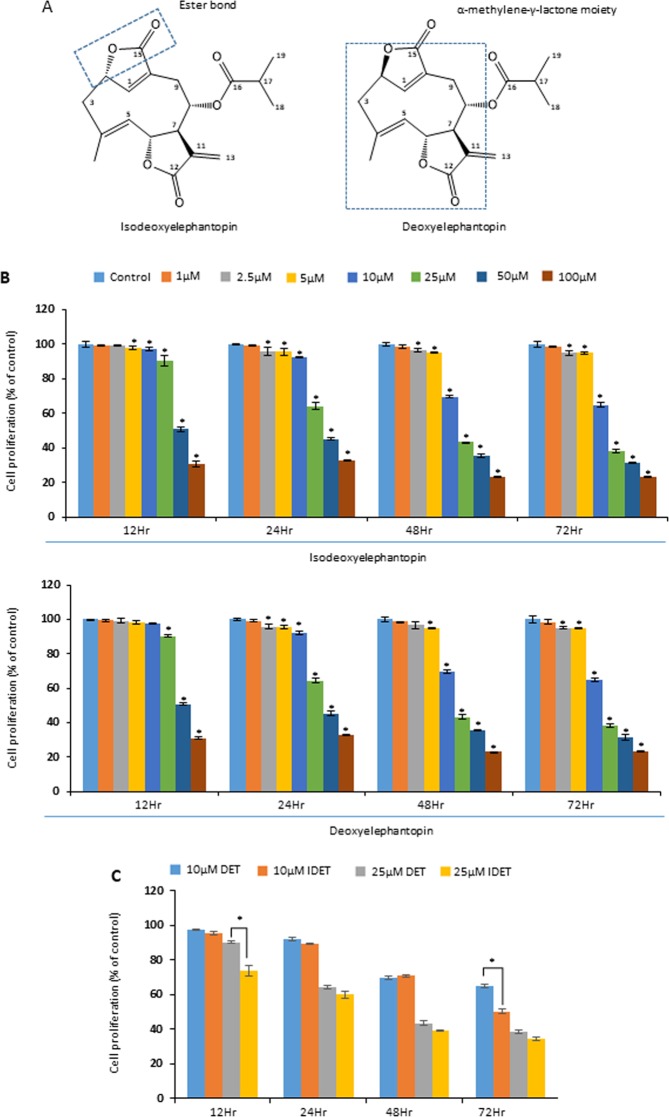
IDET is more effective in suppressing the breast cancer cells proliferation as compared to DET. (**A**) The chemical structure of IDET and DET. (**B**) MDA-MB-231 cells were exposed to different concentrations of IDET and DET for 12–72 hrs. The MTT assay was used to examine the proliferation of cells. (**C**) The relative sensitivity of MDA-MB-231 cells to 10 µM and 25 µM concentrations of two agents was examined at 12–72 hrs. The values indicate mean ± SE (3 experiments). *Shows the significance level in comparison to untreated group; *P* < 0.05. IDET, isodeoxyelephantopin; DET; deoxyelephantopin.

In this study, we examined the relative potency of IDET and DET in breast cancer cells (Fig. 1A). Preliminary experiments were performed with both IDET and DET. However, most experiments were performed with IDET. We examined the anti-tumorigenic and anti-inflammatory activities of IDET. Because of availability of multiple variants, MDA-MB-231 was used for most experiments. We also used other cell lines such as T-47D and MCF-7 to examine the IDET’s specificity. The underlying mechanism for the anti-carcinogenic activities of IDET was examined.

### IDET exhibit stronger anti-proliferative activities in breast cancer cells as compared to DET

First, the relative anti-proliferative activities of IDET and DET was examined. We exposed MDA-MB-231 cells to 1–100 µM IDET and DET for 12–72 hrs. The mitochondrial reductase activity was measured using the MTT substrate. The anti-proliferative activities of IDET was stronger as compared to DET (Fig. 1B). For example, the cell proliferation was suppressed by 36% when cells were exposed to 10 µM DET for 72 hrs (Fig. 1C). However, the cell proliferation was suppressed by 50% after exposure of cells to 10 µM IDET for 72 hrs. Similarly, a respective 10% and 27% suppression in cell proliferation was observed after exposure of cells for 12 hrs to 25 µM DET and 25 µM IDET, respectively.

Whether IDET and DET possess drug like properties was examined by *in silico* tools. Using Lipinski’s rule of five and ADMET analysis, we found that both IDET and DET exhibited similar characteristics (Table 2). The predicted lipophilicity (log P), topological polar surface area, molecular weight, hydrogen bond acceptor, hydrogen bond donor, and rotatable bonds were found to be 2.25, 78.92, 344.36, 6, 0, and 3, respectively. Furthermore, both IDET and DET were permeable to blood brain barrier and intestine without any evidence of carcinogenic and genotoxic effects.

**Table 2:** *In silico* ADMET analysis of IDET and DET.

Ligand	Absorption		Metabolism										Excretion	Toxicity	
	BBB	HIA	P-Glycoprotein		CYP-450 substrate			CYP-450 inhibitor							
			Pg-S	Pg-I 1/2	2C9	2D6	3A4	1A2	2C9	2D6	2C19	3A4	ROCT	AMES	Carcinogen
-IDET [Pubchem ID: 38359583; SMIL ID: CC1=CC2C(C(CC3=CC(C1)OC3=O)OC(=O)C(=C)C)C(=C)C(=O)O2]	+	+	−	−/+	−	−	−	−	−	−	−	−	−	−	−
DET [Pubchem ID: 6325056; SMIL ID: CC1=CC2C(C(CC3=CC(C1)OC3=O)OC(=O)C(=C)C)C(=C)C(=O)O2]	+	+	−	−/+	−	−	−	−	−	−	−	−	−	−	−

Abbreviation: IDET, Isodeoxyelephantopin; DET, Deoxyelephantopin.

Because the anti-proliferative activities of IDET was relatively stronger as compared to DET, we used IDET for most of the other experiments.

### IDET suppresses the proliferation of multiple breast cancer cells

**Figure 2:**
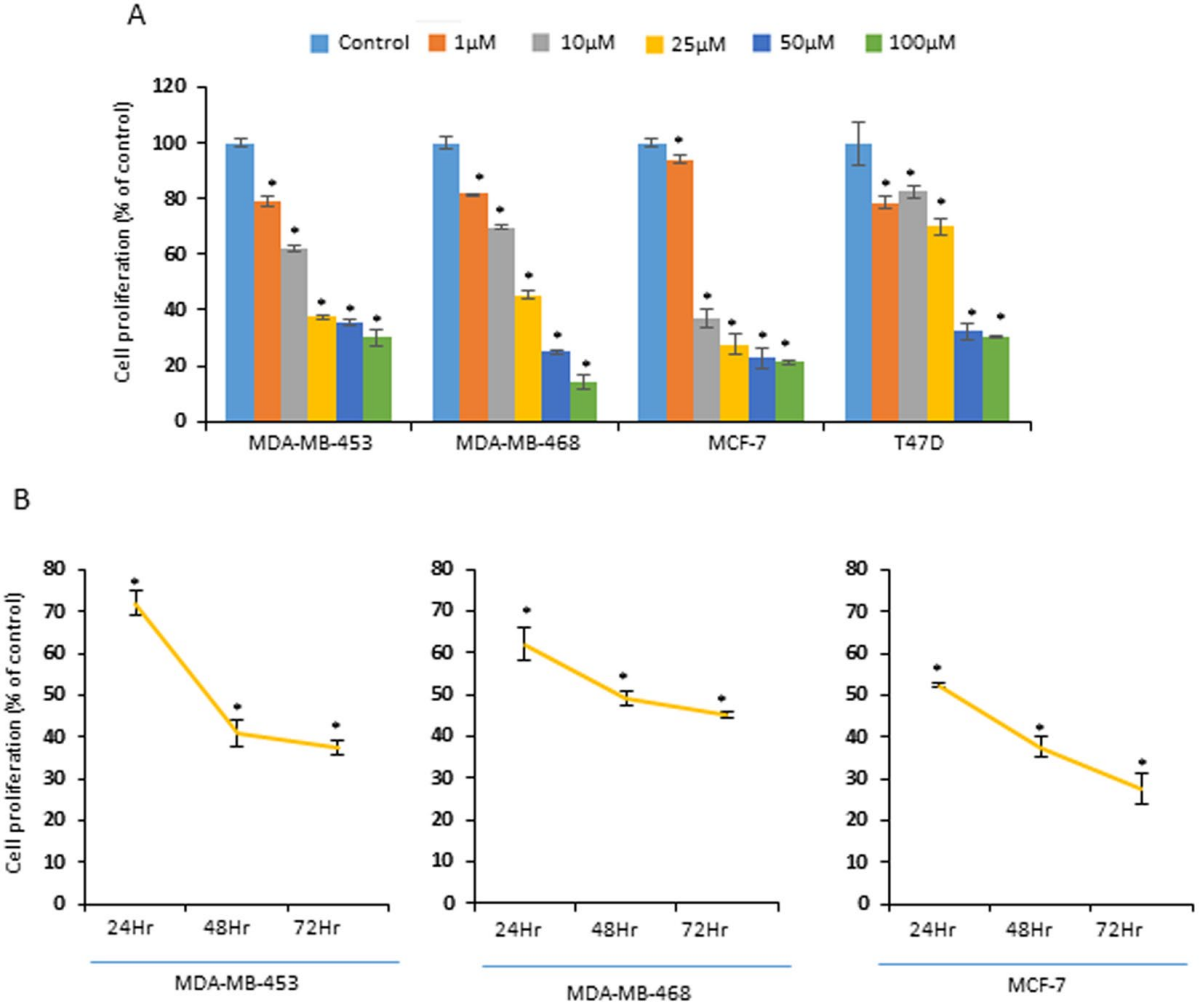
IDET suppresses the proliferation of multiple breast cancer cells in a dose- and time- dependent manner. Breast cancer cell lines (T47D, MCF-7, MDA-MB-468, MDA-MB-453) were exposed to (**A**) different concentrations of IDET for 72 hrs or (**B**) 25 µM IDET for 24–72 hrs. The proliferation of cells was assessed by measuring the mitochondrial reductase activity. The values indicate mean ± SE (3 experiments). *Shows the significance level in comparison to untreated group; *P* < 0.05. IDET, isodeoxyelephantopin.

Whether the anti-proliferative activities of IDET is specific to one cell line was examined. For this, we used multiple breast cancer cell lines. T47D, MCF-7, MDA-MB-468 and MDA-MB-453 cells were treated with 1–100 µM IDET for 72 hrs. The cell viability was decreased in a dose dependent manner (Fig. 2A). Similarly, when cells were exposed to 25 µM IDET for 24–72 hrs, the viability of cells was suppressed in a time dependent manner (Fig. 2B). These observations suggest that the effects of IDET is not cell-type specific.

### IDET suppresses the colony formation of breast cancer cells

**Figure 3:**
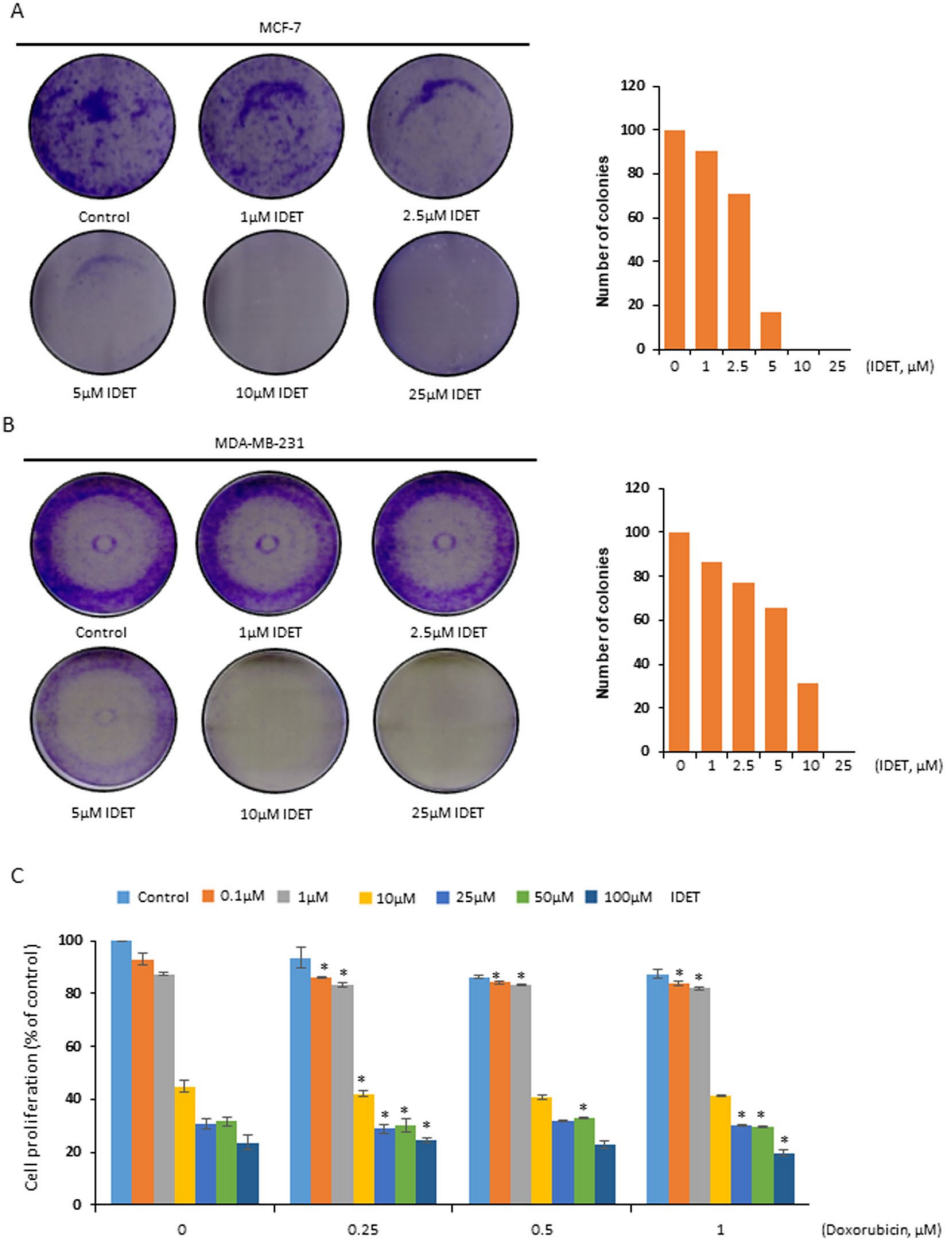
IDET suppresses the colony formation and enhances the sensitivity of breast cancer cells to doxorubicin. (**A**,**B**) MDA-MB-231 and MCF-7 cells (1000 cells/well) were exposed to different concentrations of IDET. After 24 hrs, IDET was washed off and cells were allowed to form colonies for 7 days. After staining with 0.1% crystal violet, the colonies were counted manually. IDET reduced the colonies number in a dose dependent manner. (**C**) MCF-7 cells were treated with different concentrations of IDET. After 24 hrs, IDET was washed off and cells were treated with different concentrations of doxorubicin for another 24 hrs. The combination of two agents significantly suppressed the viability of cells in comparison to individual agents. Where shown, the values are mean ± SE (3 experiments). *Shows the significance level in comparison to untreated group; *P* < 0.05. IDET, isodeoxyelephantopin.

Whether IDET affects the colony formation of breast cancer cells was examined. We exposed MCF-7 (Fig. 3A) and MDA-MB-231 (Fig. 3B) cells to 1–25 µM IDET for 24 hrs. The IDET was then washed off and the colony formation was examined after 7 days. At a concentration as low as 2.5 µM IDET, a drastic decrease in the colony formation was observed.

### Breast cancer cells are sensitized to doxorubicin by IDET

Doxorubicin is a commonly used chemotherapy for breast cancer. However, patients develop resistance over time. Whether the sensitivity of breast cancer cells to doxorubicin can be enhanced by IDET was examined. We exposed MCF-7 cells to different concentrations of IDET before treatment with doxorubicin. Both IDET and doxorubicin suppressed the viability of cancer cells (Fig. 3C). However, when cells were pretreated with IDET, the sensitivity of cells to doxorubicin was significantly increased. For example, at 0.25 µM doxorubicin, 7% reduction in the viability of cells was observed. However, the pretreatment of cells with 1 µM and 10 µM IDET before 0.25 µM doxorubicin reduced the viability by 17% and 58%, respectively.

### IDET induces apoptosis in breast cancer cells

**Figure 4:**
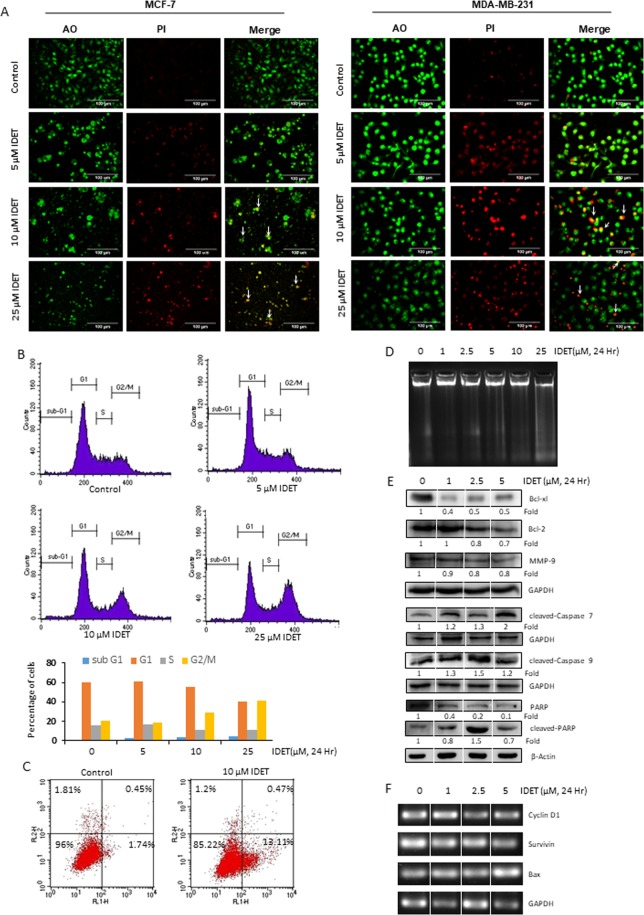
IDET induces apoptosis in breast cancer cells. (**A**) MDA-MB-231 and MCF-7 cells were treated with 5, 10 and 25 µM IDET. After 24 hrs, cells were stained with AO/PI, washed and examined under fluorescence microscope. (**B**) Untreated and IDET treated MDA-MB-231 cells were stained with PI, and flow cytometry was used to analyze the cells at different phases of cell cycle. (**C**) IDET induces PS externalization in breast cancer cells. MDA-MB-231 cells were exposed to 10 µM IDET for 24 hrs, stained with Alexafluor 488 conjugated annexin V antibody and analyzed by flow cytometry. (**D**) DNA was extracted from control and treated cells and electrophoresed on 1.5% agarose gel containing ethidium bromide. (**E**) The whole cell extract obtained from untreated and treated MDA-MB-231 cells was used to examine the expression pattern of cell survival, invasive, cleaved caspase, and PARP proteins. The corresponding GAPDH and β-Actin was used for the normalization of the data. The fold reduction in the experimental group as compared to the control group is indicated below the blot. (**F**) The RNA was extracted from the untreated and treated cells, reverse transcribed, amplified by PCR, electrophoresed on the agarose gel and stained with ethidium bromide. The blots were derived from the same gel and the data was normalized using GAPDH as an internal control. IDET, isodeoxyelephantopin.

One possibility for the reduction in the viability of cells after IDET treatment may be due to induction of apoptosis. A variety of assays were performed to examine the apoptosis inducing potential of IDET. First, AO/PI dual staining was carried out to accurately determine the cell viability. After dual staining, the live nucleated cells fluoresce green and the dead nucleated cells fluoresce red. An increase in the IDET concentration was associated with a decrease in the viability of MCF-7 cells and increased number of dead cells (Fig. 4A, *left*). A similar trend in the viability of MDA-MB-231 cells was observed after treatment with IDET (Fig. 4A, *right*). The membrane blebbing and nuclear condensation, which are characteristics of early apoptosis was also observed after IDET treatment. Next, we examined the effects of lactone on the cell cycle distribution by flow cytometry. While the percentage of cells in the sub-G1 and G2/M phase was increased, a decrease in the population of cells in the G1 and S phase was observed after IDET treatment (Fig. 4B). For example, 3.5 folds increase in the sub-G1 population was observed at 25 µM IDET in comparison to control. Similarly, 2 folds increase in the G2/M phase was observed at 25 µM IDET in comparison to control. Overall, these results suggest that IDET induces cell cycle arrest at sub-G1 and G2/M phase in MDA-MB-231 cells. A key feature of early apoptosis is the PS externalization from the inner surface to the outer surface of plasma membrane that disrupts the membrane symmetry. While Annexin V has a high affinity for PS, PI binds to DNA^35^. Thus, dual staining with Annexin V and PI can be used to distinguish cells undergoing early apoptosis and late apoptosis. In control group, 1.7% cells were stained with Annexin V. However, 13.1% Annexin V positive cells were observed at 10 µM IDET (Fig. 4C). The late stages of apoptosis are associated with cleavage of DNA into 180–200 base pair fragments known as DNA ladders. Exposure of cells to IDET induced DNA laddering in a concentration dependent manner (Fig. 4D). Next, we examined if IDET can modulate the expression of tumorigenic proteins. The expression of anti-apoptotic (Bcl-xL, Bcl-2) and invasive (MMP-9) proteins was significantly suppressed while an induction in caspase and PARP cleavage was observed by IDET (Fig. 4E). The lactone also suppressed the expression of mRNA transcript of genes involved in cell survival (survivin) and proliferation (cyclin D1) (Fig. 4F). The expression of mRNA transcript of proapoptotic Bax was also induced by IDET (Fig. 4F). Collectively, IDET can induce apoptosis in breast cancer cells.

### IDET disrupts mitochondrial membrane potential and induces ROS generation in breast cancer cells

**Figure 5:**
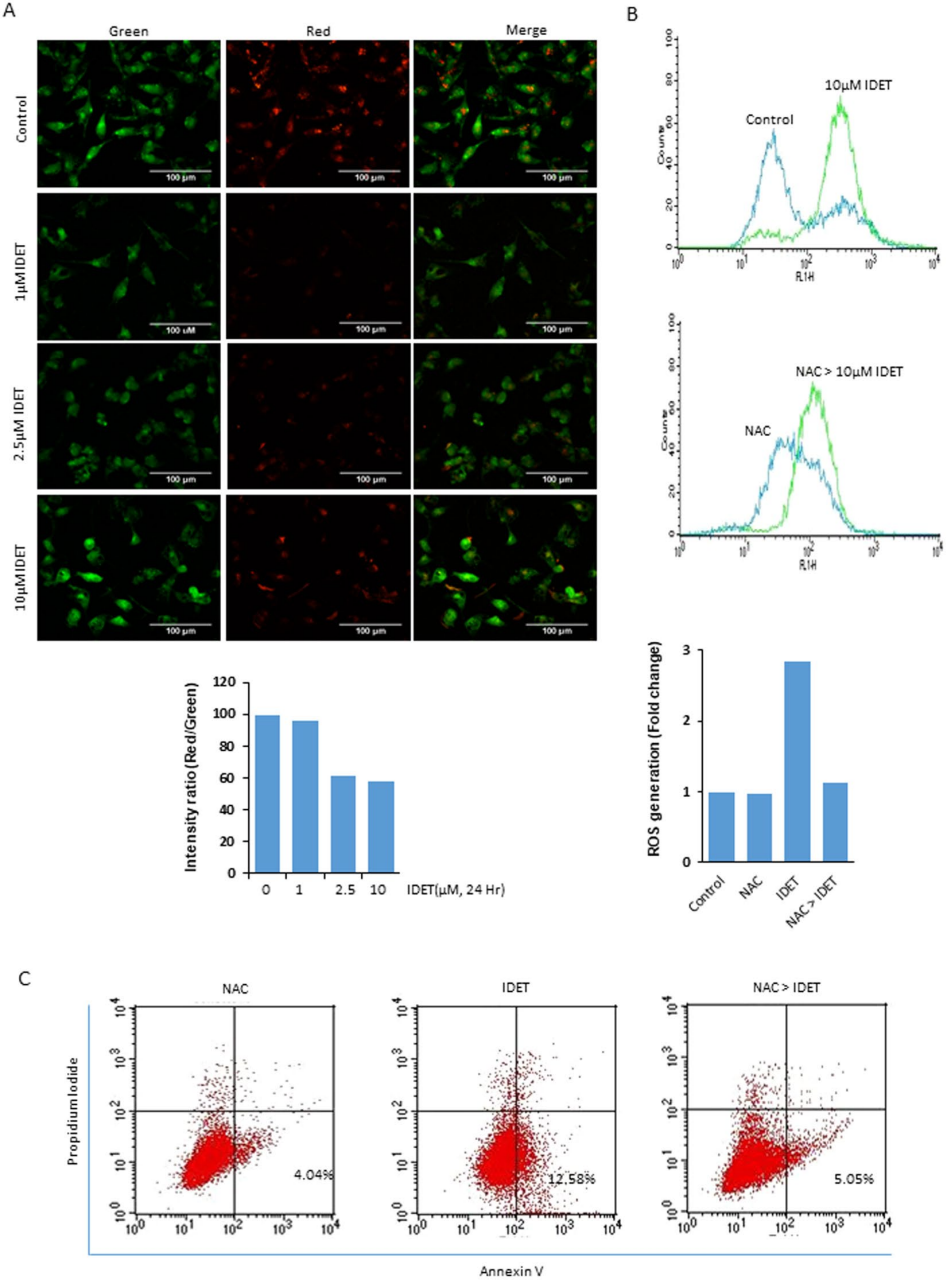
IDET disrupts mitochondrial membrane potential and induces ROS generation. (**A**) MDA-MB-231 cells were treated with 1–10 µM IDET. After 24 hrs, the cells were stained with JC-1 and examined under the fluorescence microscope. Whereas green fluorescence indicates the cells with depolarized mitochondria, red fluorescence shows cells with intact mitochondria. (**B**) Cells were treated with indicated concentrations of IDET without or with pre-treatment of NAC for 1 hr. The cells were then stained with H2DCFDA (10 µM) for 30 minutes and flow cytometry was used to measure ROS generation. (**C**) Control and treated cells were stained with Alexafluor 488 conjugated annexin V antibody and analyzed by flow cytometry for PS externalization. IDET, isodeoxyelephantopin; NAC, N-acetyl-L-cysteine.

We used the fluorochrome JC-1 to examine if IDET induced apoptosis in breast cancer cells require mitochondria. In control cells with intact mitochondria, the fluorochrome produces red fluorescence. However, with the depolarization of mitochondria and the reduction of the mitochondrial membrane potential, the intensity of the green fluorescence is increased. The staining of control cells with JC-1 produced prominent red fluorescence and minimal green fluorescence (Fig. 5A). However, the treatment of cells with IDET produced a reduction in the red fluorescence and an increase in the green fluorescence (Fig. 5A). Overall, these results suggest that IDET induces depolarization in mitochondrial membrane potential.

Whether IDET can induce ROS generation in breast cancer cells was examined. MDA-MB-231 cells were exposed to 10 µM IDET for 1 hr and ROS generation was examined by staining the cells with H2DCFDA. As shown in Fig. 5B, the treatment of cells with 10 µM IDET produced 2.8 folds increase in ROS generation. Furthermore, the use of NAC almost completely suppressed the ROS generation induced by IDET. Similarly, the PS externalization induced by IDET was also suppressed by the use of NAC (Fig. 5C). Overall, these results suggest that IDET can induce ROS generation, which is required for apoptosis induction in breast cancer cells.

### IDET reduces the migration of breast cancer cells

**Figure 6:**
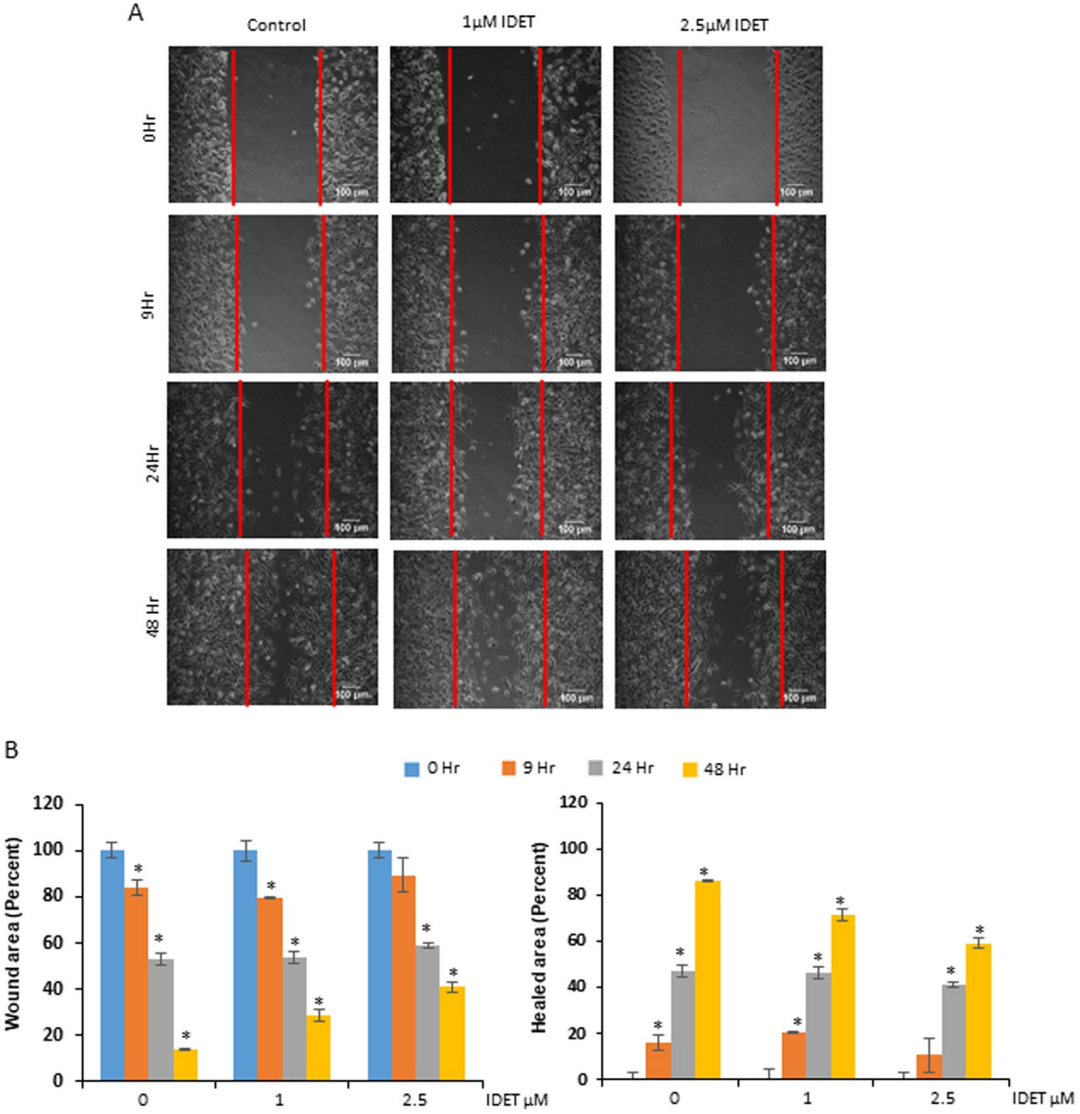
The breast cancer cells migration is suppressed by IDET. (**A**) The MDA-MB-231 cells were seeded and allowed to grow till 70% confluency. The cells were then scratched using sterile micro tip and exposed to different concentrations of IDET. (**B**,**C**) The width of the scratched area over a period of time was visualized examined under phase contrast microscope. Then, the wound size and healed area (percent) was calculated. The migration potential of MDA-MB-231 cells was decreased by IDET. Where shown, the values are mean ± SE (3 experiments). *Shows the significance level in comparison to untreated group; *P* < 0.05. IDET, isodeoxyelephantopin.

We examined if IDET can suppress the motility of breast cancer cells which is required for the invasion and metastasis. MDA-MB-231 cells were wounded at 70% confluency and cultured in the presence of IDET for 9–48 hrs. The wound area after 48 hrs in control, 1 µM and 2.5 µM IDET groups was found to be 14%, 28%, and 40%, respectively (Fig. 6A). Similarly, the wound area was significantly healed in the control cells over time. However, IDET significantly suppressed the healing potential of cancer cells. For example, the healed area after 48 hrs was found to be 85%, 71%, and 59%, in the control, 1 µM and 2.5 µM IDET groups, respectively. In conclusion, IDET can suppress the motility of breast cancer cells.

#### IDET inhibits NF-κB activation and interacts with NF-κB associated proteins

The pro-inflammatory transcription factor, NF-κB plays a crucial role in the survival, proliferation, migration and chemoresistance of breast cancer cells. Whether, the lactone can reverse the NF-κB activation induced by okadaic acid (OA) was examined. We treated MDA-MB-231 cells either with OA and IDET alone or with IDET followed by OA. The NF-κB p65 cellular localization was examined by immunocytochemistry. An induction in the p65 nuclear translocation was observed after the treatment of cells with OA. IDET alone did not affect the localization of p65. However, by pretreatment of cells with IDET, OA induced p65 nuclear translocation was significantly suppressed.

**Figure 7:**
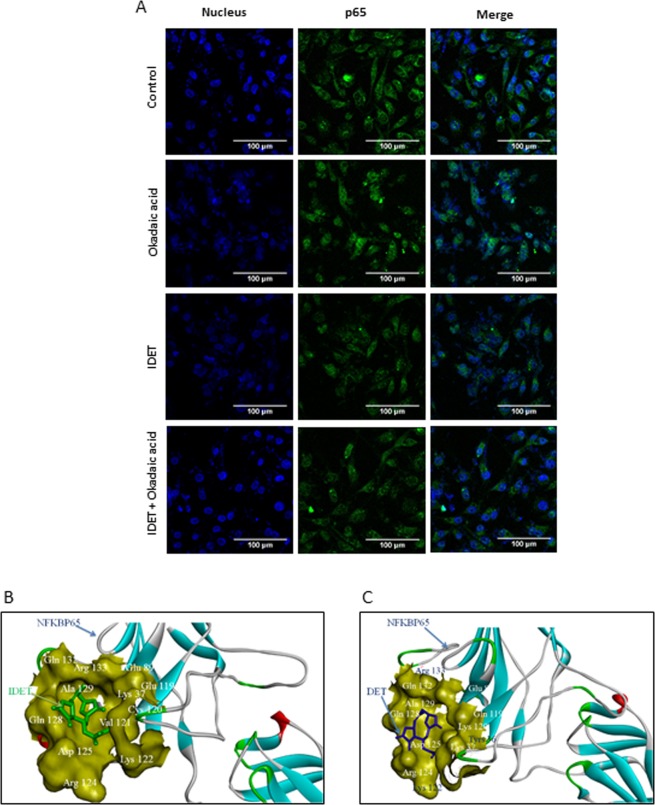
IDET suppresses nuclear translocation of NF-κB-p65 and interacts with NF-κB-p65. (**A**) Cells were treated with 10 µM IDET. After 6 hrs, IDET was removed and cells were cultured in the presence of 100 nM okadaic acid for 4 hrs. Immunocytochemistry was used to examine localization of NF-kB p65. Note a suppression in OA induced p65 nuclear translocation after IDET treatment. (**B**,**C**) Molecular docking for the interaction of p65 with IDET and DET.

Whether IDET and DET interact with NF-κB associated proteins was examined by *in silico* tools. The binding energies (kcal/mol) and Ki (dissociation constant, µM) values of p65, p50, inhibitor of kappa-B alpha (IκBα), transforming growth factor-β-activating kinase-1 (TAK-1) and IκB kinase alpha (IKKα) with IDET were −6.67 and 12.86, −6.23 and 27.05, −5.19 and 156, −6.25 and 26.22, −6.15 and 30.89, respectively (Table 3). Similarly, the binding energies (kcal/mol) and Ki (µM) values of p65, p50, IκBα, TAK-1 and IKKα with DET were in a respective order of −6.51 and 16.99, −6.37 and 21.29, −4.46 and 539.25, −6.71 and 12.10, −5.81 and 54.83. The affinity of p65 with IDET (binding energy: −6.67 kcal/mol; Ki: 12.86 µM; 3 hydrogen bonds) was stronger as compared to DET (binding energy: −6.51 kcal/mol; Ki: 16.99 µM; 2 hydrogen bonds) (Fig. 7B,C). A similar pattern was observed for IκBα and IKKα. However, TAK-1 exhibited higher affinity for DET as compared to IDET (Table 3).

**Table 3:** Molecular docking analyses of IDET and DET with major proteins of NF-κB signaling pathway.

Receptor	Binding Energy (kcal/mol)		Ki (µM)		Binding residues		H-Bonds	Distance (Å)	H-Bonds	Distance (Å)
	IDET	DET	IDET	DET	IDET	DET	IDET		DET	
p65 (PDB: 1NFI)	−6.67	−6.51	12.86	16.99	Lys37, Pro87, Glu89, Gln119, Cys120, Val121, Lys122, Arg124, Asp125, Gln128, Ala129, Arg133	Tyr36, Lys37, Glu89, Gln119, Cys120, Val121, Lys122, Arg124, Asp125, Gln128, Ala129, Gln132, Arg133	CYS120:N-IDET1:O	3.15151	LYS122:N-DET:O	2.63244
							ARG124:NH1-IDET1:O	2.84434	ARG124:NH2-DET:O	3.17358
							ARG124:NH2-IDET1:O	2.96715		
p50 (PDB: 1NFI)	−6.23	−6.37	27.05	21.29	Gly266, Trp295, Gly297, Phe298, Asp300, Lys315, Thr316, Pro317, Lys318	Thr264, Gly266, Glu296, Gly297, Phe298, Gly299, Asp300, Thr316, Pro317, Lys318	PHE298:N-IDET:O	2.71851	PHE298:N-DET:O	3.19342
							LYS318:N-IDET:O	2.92484	LYS318:N-DET:O	2.93974
IkBα (PDB: 1NFI)	−5.19	−4.46	156.00	539.25	Ser191, Ile192, His193, Gly194, Tyr195, Leu227, Asn229	Glu153, Asn180, Asn182, His184, Leu189, Ile192, His193, Leu223, Leu227	ASN229:ND2-IDET:O	3.16329	ASN182:ND2-DET:O	2.85601
									ASN182:ND2-DET:O	3.20524
TAK-1 (PDB: 2EVA)	−6.25	−6.71	26.22	12.10	Val42, Gly43, Arg44, Gly45, Ala46, Val50, Ser111, Tyr113, Asn114, Pro160, Asn161, Asp175	Val42, Gly43, Arg44, Gly45, Val50, Gly110, Ser111, Tyr113, Asn114, Pro160, Leu163	SER111:OG-IDET:O	2.84141	GLY45:N-DET:O	3.00685
							SER111:OG:B-IDET1:O	2.82763	ASN114:ND2:B-DET:O	3.06489
IKKα (PDB: 5TQY)	−6.15	−5.81	30.89	54.83	Glu118, Leu122, Asn309, Leu310, Lys311, Ile312, Val313, His314, Met383	Glu118, Leu310, Lys311, Ile312, Val313, His314, Met383	ILE312:N-IDET:O	3.15137	ILE312:N-DET:O	3.11097
							HIS314:N-IDET:O	2.73251	ILE312:N-DET:O	2.8978
							MET383:N-IDET:O	3.10888	HIS314:N-DET:O	2.75596

Abbreviation: IDET, Isodeoxyelephantopin (PubChem ID: 38359583); DET, Deoxyelephantopin (PubChem ID: 6325056).

### IDET modulates the lncRNAs expression in breast cancer cells

**Figure 8:**
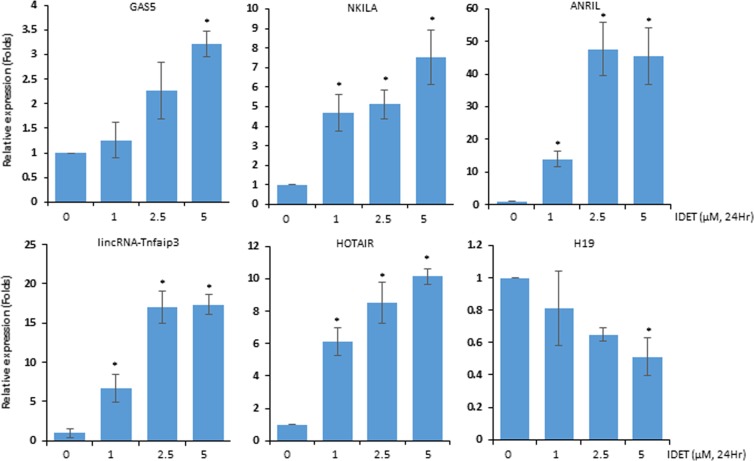
The lncRNA expression in breast cancer cells is modulated by IDET. MDA-MB-231 cells were exposed to indicated concentrations of IDET for 24 hrs. RNA was isolated, cDNA was synthesized and the lncRNAs expression pattern was assessed by quantitative RT-PCR. The oncogenic H19 expression was reduced while the tumor suppressive lncRNAs (GAS5, NKILA) expression was increased. *Shows the significance level in comparison to untreated group; *P* < 0.05.

The lncRNAs are known to modulate multiple steps of tumor development. Some lncRNAs such as NKILA and H19 can also cross talk with NF-κB. Whether the expression of lncRNAs is modulated by IDET was examined. The lactone up-regulated the expression of growth arrest specific 5 (GAS5), NKILA, ANRIL, tumor necrosis factor α-induced protein 3 (lincRNA-Tnfaip3) and HOTAIR in a concentration dependent manner (Fig. 8). For example, the expression of GAS5 was induced by 1.3 folds, 2.3 folds and 3.2 folds at 1, 2.5 and 5 µM IDET, respectively. Similarly, a respective 4.7 folds, 5.1 folds and 7.5 folds increase in NKILA expression was observed by 1, 2.5 and 5 µM IDET, respectively. Conversely, H19 expression was reduced by IDET in a concentration dependent manner. Collectively, IDET can modulate the lncRNAs expression pattern in breast cancer cells.

## Discussion

The multigenic breast cancer is the leading cause of cancer associated deaths in women globally. The pro-inflammatory NF-κB is known to regulate multi-steps of breast tumor development and chemoresistance. Therefore, the suppression of NF-κB activation can prevent or delay the onset of breast tumor development. The Mother Nature has been a gold mine for the discovery of anti-cancer agents. Roughly 50% of the anticancer agents approved between 1940 and 2014 were derived from Nature. In this study, we examined the activities of sesquiterpene lactones such as IDET and DET against breast cancer. The molecular mechanism by which the lactones exhibit activities was also examined.

IDET was more effective in reducing the proliferation of breast cancer cells as compared to DET. Both the lactones contain α-methylene-γ-lactone which can contribute to their cytotoxic activities^36^. The γ-lactone ring oxygen atom at C-2 is in β-orientation and α-orientation in DET and IDET, respectively. The presence of C_11_-C_13_ exocyclic methylene in conjugation with γ-lactone can contribute to the cytotoxicity of both IDET and DET. In agreement with these observations, Helenain, a sesquiterpene lactone with reactive α, β-unsubstituted cyclopentenone ring has been reported to exhibit potent cytotoxicity in cancer cell lines^37^. Why IDET is more potent than DET remains to be elucidated.

Apoptosis and necrosis are two common modes of cell death. While apoptosis is programmed mode of cell death, necrosis may result in inflammation which is a precursor to several chronic diseases including cancer^38^. Therefore, agents with potential to selectively induce apoptosis in cancer cells are preferred^39^. The presence of membrane blebbing, nuclear condensation, phosphatidylserine externalization and DNA laddering by IDET suggest the potential of this lactone in inducing apoptosis in breast cancer cells. We observed that IDET suppresses the expression of cell survival (Bcl-xL, Bcl-2) and invasive (MMP-9) proteins. Furthermore, an induction in caspase cleavage was observed by IDET. Additionally, the lactone reduces the expression of mRNA transcripts of genes involved in survival (survivin) and proliferation (cyclin D1). The lactone also induces the expression of proapoptotic gene, Bax. The early phases of apoptosis is associated with phosphatidylserine externalization, disruption of mitochondrial membrane potential (ΔΨ), insertion of proapoptotic proteins into the membrane and the cytochrome c release from mitochondria to the cytoplasm^40^. The ability of IDET to induce loss in ΔΨ suggest that the mitochondria is involved in the apoptosis induction by this lactone. NF-κB is known to regulate the expression of over 500 tumorigenic genes and proteins. The inhibitory effects of IDET on the tumorigenic genes and proteins may be due to the negative regulation of NF-κB activation pathway by this lactone. The mechanism of NF-κB inactivation by IDET in breast cancer cells was not examined. However, a previous study demonstrated the inhibitory effects of IDET on IKK, a central kinase in the NF-κB signaling pathway^22^. It is likely that the reduction in the NF-κB activity by IDET in breast cancer cells is due to its inhibitory effects on IKK activity.

Cancer cells are normally characterized by dysregulation in cell cycle^41^. Thus, targeting cell cycle could be a potential strategy for breast cancer therapy. We observed an accumulation of cells in the G2/M phase. Similar to these observations, IDET has been demonstrated to induce G2/M cell cycle arrest in A549 lung carcinoma cells^42^. Previous studies have demonstrated that Cdc2/Cyclin B1 complex regulate G2/M cell cycle arrest under oxidative stress^43^. IDET was found to induce ROS generation in breast cancer cells that was reversed by NAC. The chemotherapeutic agents and phytochemicals work through generation of ROS^44^. The mechanistic association between ROS generation and G2/M cell cycle arrest by IDET remains to be elucidated.

The cancer cell migration is an important step prior to the invasion and metastasis. The majority of the breast cancer deaths are because of the potential of the tumor to metastasize to other organs. In our observations, IDET significantly reduced the migration of MDA-MB-231 cells. Consistence with these observations, the expression of MMP-9, an invasive protein regulated by NF-κB was also suppressed. The reduction of NF-κB activation by IDET may be responsible for the suppression in MMP-9 expression and the inhibition of migration of MDA-MB-231 cells.

Dysregulation in the lncRNAs expression plays a crucial role in several human malignancies including breast cancer^45^. An increase in the GAS5 and NKILA expression was observed after IDET treatment. Originally identified in breast cancer and located in the cytoplasm, NKILA masks the phosphorylation motifs of IκB by interacting with p65-IκB subunits thereby suppressing NF-κB activation^46^. While NKILA is abundantly expressed in the normal breast epithelia, its low expression associates with breast cancer metastasis and poor patient prognosis. Further, the gene silencing of NKILA results in significant phosphorylation and degradation of IκB leading to enhanced NF-κB activation^46^. An up-regulation in NKILA expression by IDET may suppress IκB phosphorylation and NF-κB activation. Similar to these observations, the anti-invasive activities of TGF-β^47^ and anti-carcinogenic activities of baicalein^48^ are mediated through NKILA.

GAS5 is known to function as tumor suppressor in a number of cancer types^49^. This lncRNA can also induce apoptosis and reduce the tumor cells proliferation^50^. GAS5 is frequently decreased in the breast cancer tissues as compared to the adjacent non-tumor tissues^51^. The chemo-resistant breast cancer cells also exhibit significantly lower expression of GAS5^51^. The development of chemoresistance by tumor cells is a major roadblock to cancer therapy. In our observations, IDET enhanced the breast cancer cells sensitivity to doxorubicin. While GAS5 expression was low under normal conditions in breast cancer, its expression was significantly upregulated by IDET. The upregulation in GAS5 expression by IDET may be responsible for its anti-proliferative, apoptosis inducing and chemosensitization activities. The chemosensitization properties of IDET are significant as the breast cancer cells develop resistance to chemotherapeutic drugs over time. Similar to these observations, downregulation in GAS5 expression decreases the therapeutic effects of dendrosomal curcumin in breast cancer cells^52^. Similarly, gambogic acid induced GAS5 expression can produce pro-apoptotic effects in bladder cancer cells^53^. The oncogenic H19 is constitutively expressed in multiple cancer types including breast cancer^28,54^. H19 expression also correlate with NF-κB activation^54^ and paclitaxel resistance^55^. The suppression in H19 expression by IDET may be another possibility for the inhibition of NF-κB activation and sensitization of breast cancer cells to doxorubicin. The oncogenic role of ANRIL and HOTAIR is reported in the breast cancer model^56^. An upregulation in the expression of ANRIL and HOTAIR by IDET may be a compensatory mechanism in response to the suppressed expression of other oncogenic lncRNAs and upregulation of tumor suppressor lncRNAs. Although the lincRNA-Tnfaip3 is an early response gene controlled by NF-κB in murine macrophages^57^, its role in the breast cancer model remains to be elucidated.

The *In Silico* data revealed the drug like properties of both IDET and DET. The Lipinski’s ‘rule of five’ suggest that most drug-able compounds have molecular weight ≤500, LogP ≤ 5, number of hydrogen bond donors ≤5, and number of hydrogen bond acceptors ≤10^58^. Both IDET and DET obeyed these rules. The pharmacokinetics analyses revealed that both lactones can cross the blood brain barrier and have better intestinal absorption. The lack of any evidence for carcinogenic and genotoxic properties further support that both lactones can be good drug candidate.

In conclusion, IDET exhibit anti-carcinogenic, pro-apoptotic and anti-proliferative activities in breast cancer cells. Further, IDET is potent as compared to DET. The NF-κB inactivation and the modulation in lncRNA expression may be underlying mechanism for the anti-carcinogenic activities of IDET. However, whether NF-κB regulate lncRNA expression or later regulate the former in response to IDET is unclear. Whether the micromolar concentrations of IDET used in the current study are physiologically relevant remains to be elucidated. However, a previous study demonstrated that IDET exhibit anti-inflammatory activities only in cancer cells but not in normal lymphocyte^59^. Future studies should examine the activities of the lactone in the breast cancer animal model. The thorough pharmacokinetics and pharmacodynamics studies in animal models should also be performed before IDET can be tested in humans by clinical trial.

## References

[1] Sahu A, Singhal U, Chinnaiyan AM (2015). Long noncoding RNAs in cancer: from function to translation. *Trends*. Cancer.

[2] Chandra Gupta S, Nandan Tripathi Y (2017). Potential of long non‐coding RNAs in cancer patients: From biomarkers to therapeutic targets. International journal of cancer.

[3] Van Grembergen O (2016). Portraying breast cancers with long noncoding RNAs. Science advances.

[4] Jin S (2019). p53-targeted lincRNA-p21 acts as a tumor suppressor by inhibiting JAK2/STAT3 signaling pathways in head and neck squamous cell carcinoma. Molecular cancer.

[5] Sen R, Baltimore D (1986). Inducibility of κ immunoglobulin enhancer-binding protein NF-κB by a posttranslational mechanism. Cell.

[6] Dai XL, Zhou SL, Qiu J, Liu YF, Hua H (2012). Correlated expression of Fas, NF-kappaB, and VEGF-C in infiltrating ductal carcinoma of the breast. Eur J Gynaecol Oncol.

[7] Nehra R (2010). BCL2 and CASP8 regulation by NF-κB differentially affect mitochondrial function and cell fate in antiestrogen-sensitive and-resistant breast cancer cells. The FASEB Journal.

[8] Gupta, S. C. *et al*. Long non-coding RNAs and nuclear factor-kB crosstalk in cancer and other human diseases. *Biochimica et Biophysica Acta-Reviews on Cancer* in press (2019). 10.1016/j.bbcan.2019.188316 PMC7775411 31639408

[9] Zhou X (2016). Long non-coding RNA ANRIL regulates inflammatory responses as a novel component of NF-κB pathway. RNA biology.

[10] Özeş AR (2017). Therapeutic targeting using tumor specific peptides inhibits long non-coding RNA HOTAIR activity in ovarian and breast cancer. Scientific reports.

[11] Zgheib C, Hodges MM, Hu J, Liechty KW, Xu J (2017). Long non-coding RNA Lethe regulates hyperglycemia-induced reactive oxygen species production in macrophages. PLOS one.

[12] Juan C (2018). The LncRNA MALAT1 regulates CD80 transcription via the NF-κB signaling pathway in the A549 cell line. Biochemical and biophysical research communications.

[13] Liu B (2015). A cytoplasmic NF-κB interacting long noncoding RNA blocks IκB phosphorylation and suppresses breast cancer metastasis. Cancer cell.

[14] Liao Z, Zhao J, Yang Y (2018). Downregulation of lncRNA H19 inhibits the migration and invasion of melanoma cells by inactivating the NF-κB and PI3K/Akt signaling pathways. Molecular medicine reports.

[15] Newman DJ, Cragg GM (2012). Natural products as sources of new drugs over the 30 years from 1981 to 2010. Journal of natural products.

[16] Hiradeve SM, Rangari VD (2014). Elephantopus scaber Linn.: A review on its ethnomedical, phytochemical and pharmacological profile. Journal of applied biomedicine.

[17] Yam MF (2009). Anti-inflammatory and analgesic effects of Elephantopus tomentosus ethanolic extract. Journal of acupuncture and meridian studies.

[18] Chan C, Chan G, Awang K, Abdul Kadir H (2016). Deoxyelephantopin from elephantopus scaber inhibits HCT116 human colorectal carcinoma cell growth through apoptosis and cell cycle arrest. Molecules.

[19] Liang Q-L, Min Z-D, Tang Y-P (2008). A new elemanolide sesquiterpene lactone from Elephantopus scaber. Journal of Asian natural products research.

[20] Wang Y (2017). Isodeoxyelephantopin induces protective autophagy in lung cancer cells via Nrf2-p62-keap1 feedback loop. Cell death & disease.

[21] Yan GR (2013). Quantitative proteomics characterization on the antitumor effects of isodeoxyelephantopin against nasopharyngeal carcinoma. Proteomics.

[22] Ichikawa H (2006). Isodeoxyelephantopin, a novel sesquiterpene lactone, potentiates apoptosis, inhibits invasion, and abolishes osteoclastogenesis through suppression of nuclear factor-κB (NF-κB) activation and NF-κB-regulated gene expression. Clinical Cancer Research.

[23] Gupta SC (2011). Bharangin, a diterpenoid quinonemethide, abolishes constitutive and inducible nuclear factor-kappaB (NF-kappaB) activation by modifying p65 on cysteine 38 residue and reducing inhibitor of nuclear factor-kappaB alpha kinase activation, leading to suppression of NF-kappaB-regulated gene expression and sensitization of tumor cells to chemotherapeutic agents. Mol Pharmacol.

[24] Gupta SC (2013). Nimbolide, a limonoid triterpene, inhibits growth of human colorectal cancer xenografts by suppressing the proinflammatory microenvironment. Clin Cancer Res.

[25] Herrmann M (1994). A rapid and simple method for the isolation of apoptotic DNA fragments. Nucleic Acids Res.

[26] Bognar Z (2017). Desethylamiodarone-A metabolite of amiodarone-Induces apoptosis on T24 human bladder cancer cells via multiple pathways. PLoS One.

[27] Gupta SC (2016). Regulation of breast tumorigenesis through acid sensors. Oncogene.

[28] Awasthee N (2018). Anti-cancer activities of Bharangin against breast cancer: Evidence for the role of NF-κB and lncRNAs. Biochimica et Biophysica Acta (BBA)-General Subjects.

[29] Gupta SC, Singh R, Pochampally R, Watabe K, Mo Y-Y (2014). Acidosis promotes invasiveness of breast cancer cells through ROS-AKT-NF-κB pathway. Oncotarget.

[30] Zhang A (2013). The human long non-coding RNA-RoR is a p53 repressor in response to DNA damage. Cell Res.

[31] Schmittgen TD, Livak KJ (2008). Analyzing real-time PCR data by the comparative C(T) method. Nat Protoc.

[32] Cheng F (2012). admetSAR: a comprehensive source and free tool for assessment of chemical ADMET properties. J Chem Inf Model.

[33] Morris GM (2009). AutoDock4 and AutoDockTools4: Automated docking with selective receptor flexibility. J Comput Chem.

[34] Dhasmana A (2016). Titanium dioxide nanoparticles provide protection against polycyclic aromatic hydrocarbon BaP and chrysene-induced perturbation of DNA repair machinery: A computational biology approach. Biotechnol Appl Biochem.

[35] Koopman G (1994). Annexin V for flow cytometric detection of phosphatidylserine expression on B cells undergoing apoptosis. Blood.

[36] Ghantous A, Gali-Muhtasib H, Vuorela H, Saliba NA, Darwiche N (2010). What made sesquiterpene lactones reach cancer clinical trials?. Drug discovery today.

[37] Woerdenbag HJ (1994). Cytotoxicity of flavonoids and sesquiterpene lactones from Arnica species against the GLC4 and the COLO 320 cell lines. Planta Medica.

[38] Schwartzman RA, Cidlowski JA (1993). Apoptosis: the biochemistry and molecular biology of programmed cell death. Endocrine reviews.

[39] Hanahan D, Weinberg RA (2000). The hallmarks of cancer. cell.

[40] Armstrong JS (2006). The role of the mitochondrial permeability transition in cell death. Mitochondrion.

[41] Ruiz-Casado A (2017). Exercise and the hallmarks of cancer. Trends in cancer.

[42] Kabeer FA (2013). Antineoplastic effects of deoxyelephantopin, a sesquiterpene lactone from Elephantopus scaber, on lung adenocarcinoma (A549) cells. Journal of integrative medicine.

[43] Bunz F (1998). Requirement for p53 and p21 to sustain G2 arrest after DNA damage. Science.

[44] Schumacker PT (2006). Reactive oxygen species in cancer cells: live by the sword, die by the sword. Cancer cell.

[45] Collette J, Le Bourhis X, Adriaenssens E (2017). Regulation of human breast cancer by the long non-coding RNA H19. International journal of molecular sciences.

[46] Su F, Li D, Zeng M, Song E (2015). A cytoplasmic NF-kB interacting long noncoding RNA blocks IkB phosphorylation and suppresses breast cancer metastasis. Cancer Cell.

[47] Lu Z (2018). TGF-β-induced NKILA inhibits ESCC cell migration and invasion through NF-κB/MMP14 signaling. Journal of Molecular Medicine.

[48] Yu X (2018). Baicalein inhibits breast cancer growth via activating a novel isoform of the long noncoding RNA PAX8‐AS1‐N. Journal of cellular biochemistry.

[49] Mourtada-Maarabouni M, Pickard M, Hedge V, Farzaneh F, Williams G (2009). GAS5, a non-protein-coding RNA, controls apoptosis and is downregulated in breast cancer. Oncogene.

[50] Pickard M, Williams G (2015). Molecular and cellular mechanisms of action of tumour suppressor GAS5 LncRNA. Genes.

[51] Ding Y, Duan K, Chen S (2017). Low expression of lncRNA-GAS5 promotes epithelial-mesenchymal transition of breast cancer cells *in vitro*. Nan fang yi ke da xue xue bao= Journal of Southern Medical University.

[52] Esmatabadi MJD, Motamedrad M, Sadeghizadeh M (2018). Down-regulation of lncRNA, GAS5 decreases chemotherapeutic effect of dendrosomal curcumin (DNC) in breast cancer cells. Phytomedicine.

[53] Wang M (2018). Long noncoding RNA GAS5 promotes bladder cancer cells apoptosis through inhibiting EZH2 transcription. Cell death & disease.

[54] Sun Y (2017). Knockdown of long non-coding RNA H19 inhibits multiple myeloma cell growth via NF-κB pathway. Scientific reports.

[55] Si X (2016). LncRNA H19 confers chemoresistance in ERα-positive breast cancer through epigenetic silencing of the pro-apoptotic gene BIK. Oncotarget.

[56] Ding W, Ren J, Ren H, Wang D (2017). Long noncoding RNA HOTAIR modulates MiR-206-mediated Bcl-w signaling to facilitate cell proliferation in breast cancer. Scientific reports.

[57] Ma S (2016). A long noncoding RNA, lincRNA-Tnfaip3, acts as a coregulator of NF-κB to modulate inflammatory gene transcription in mouse macrophages. The FASEB Journal.

[58] Lipinski CA, Lombardo F, Dominy BW, Feeney PJ (2001). Experimental and computational approaches to estimate solubility and permeability in drug discovery and development settings. Adv Drug Deliv Rev.

[59] Kabeer FA (2014). Isodeoxyelephantopin from Elephantopus scaber (Didancao) induces cell cycle arrest and caspase-3-mediated apoptosis in breast carcinoma T47D cells and lung carcinoma A549. cells. Chinese medicine.

